# CRISPR sequences are sometimes erroneously translated and can contaminate public databases with spurious proteins containing spaced repeats

**DOI:** 10.1093/database/baaa088

**Published:** 2020-11-18

**Authors:** Alejandro Rubio, Pablo Mier, Miguel A Andrade-Navarro, Andrés Garzón, Juan Jiménez, Antonio J Pérez-Pulido

**Affiliations:** Centro Andaluz de Biologia del Desarrollo (CABD, UPO-CSIC-JA). Facultad de Ciencias Experimentales (Área de Genética), Universidad Pablo de Olavide, Ctra. Utrera, Km.1, 41013, Sevilla, Spain; Faculty of Biology, Johannes Gutenberg University Mainz, Gresemundweg 2, 55128, Mainz, Germany; Faculty of Biology, Johannes Gutenberg University Mainz, Gresemundweg 2, 55128, Mainz, Germany; Centro Andaluz de Biologia del Desarrollo (CABD, UPO-CSIC-JA). Facultad de Ciencias Experimentales (Área de Genética), Universidad Pablo de Olavide, Ctra. Utrera, Km.1, 41013, Sevilla, Spain; Centro Andaluz de Biologia del Desarrollo (CABD, UPO-CSIC-JA). Facultad de Ciencias Experimentales (Área de Genética), Universidad Pablo de Olavide, Ctra. Utrera, Km.1, 41013, Sevilla, Spain; Centro Andaluz de Biologia del Desarrollo (CABD, UPO-CSIC-JA). Facultad de Ciencias Experimentales (Área de Genética), Universidad Pablo de Olavide, Ctra. Utrera, Km.1, 41013, Sevilla, Spain

## Abstract

The genomics era is resulting in the generation of a plethora of biological sequences that are usually stored in public databases. There are many computational tools that facilitate the annotation of these sequences, but sometimes they produce mistakes that enter the databases and can be propagated when erroneous data are used for secondary analyses, such as gene prediction or homology searching. While developing a computational gene finder based on protein-coding sequences, we discovered that the reference UniProtKB protein database is contaminated with some spurious sequences translated from DNA containing clustered regularly interspaced short palindromic repeats. We therefore encourage developers of prokaryotic computational gene finders and protein database curators to consider this source of error.

## Introduction

The genomics era has allowed a burst in the sequencing of complete genomes (1). These biological sequences are processed and analyzed with the assistance of a variety of computational tools. After genome assembly, prediction of genes is one of the first steps. This procedure is computationally easy in prokaryotes, since their genomes are approximately 90% protein-coding, and intergenic regions are relatively short (2–4). For this reason, computational gene finders predict prokaryotic genes by considering Open Reading Frames (ORFs) with a minimal length between start and stop signals of the different reading frames.

Public databases store and share the sequences of proteins putatively encoded by the predicted genes, which allows undertaking further secondary analyses and experiments. Unfortunately, databases are often contaminated with both erroneous sequences and wrong functional annotation associated data (5–7). For example, the predicted proteomes derived from complete genomes sometimes include contaminant sequences that come from the sequencing process or from the environment (8, 9).

UniProtKB is the reference protein database and maintains two different sections regarding sequence reliability (10). The Swiss-Prot section has protein sequences for which database curators associate reviewed literature and perform specific computational analyses, and the TrEMBL section contains sequences automatically annotated and not reviewed by curators. However, both sections are prone to the inclusion of spurious sequences. Known sources of erroneous protein sequences in UniProtKB are protein sequences that come from spurious ORFs inside ribosomal RNA genes (11) and proteins originating from genome assembly errors due to DNA tandem repeats (12). This is not only a problem from this protein database, and others such as NCBI RefSeq, which is one of the information sources of the two UniProtKB sections, share the same contamination (13).

While investigating the use of protein databases for gene prediction, we discovered that clustered regularly interspaced short palindromic repeats (CRISPR)-Cas systems are now another source of spurious protein sequences in protein databases. They constitute an acquired immunity system in prokaryotes composed by a series of CRISPR-associated protein-coding genes (*cas*), followed by clustered regularly interspaced short (20–60 bp long) palindromic repeats (CRISPR) that flank heterogeneous sequences (spacers) of similar length between every pair of repeats (14). Sequences from CRISPR regions do not encode proteins, but automatic gene predictions can find spurious ORFs inside them. Some gene finders prevent the annotation of new protein-coding genes when predictions overlap with non-coding sequences, including CRISPR loci (3). And specific CRISPR-Cas finders are more sensitive in the discovery of these sequences, but they work independently of more exhaustive gene finders (15, 16). This causes that erroneously annotated protein sequences sometimes enter the public databases.

These spurious protein sequences can eventually give significant similarities in homology searches, making the initial problem even worse. Here we report the importance of these misannotations to encourage developers of both gene prediction methods and protein databases to account for this source of error.

## Material and methods

### Databases

Swiss-Prot and TrEMBL entries from UniProtKB version 2019_12 (December 2019) were downloaded for archaeal and bacterial species (10). The Genomes Online Database (GOLD) was used to obtain the number of sequenced genomes by year (1). The CRISPRCasdb database was used to obtain CRISPR repeats (17) found by CRISPRCasFinder with evidence level 4, which represent the most reliable ones(15). Pfam domains (18) and creation dates were extracted from the UniProt records.

### Implementation

Several scripts written in Perl language have been developed and stored in a GitHub repository (https://github.com/UPOBioinfo/crispr_spurious/). All executions were performed in the C3UPO HPC cluster (Pablo de Olavide University, Seville, Spain), using a node with 24 cores, and globally lasted approximately a week.

### Finding of spurious proteins using already-annotated repeats

To find UniProtKB protein sequences originating from spuriously translated CRISPR sequences, we translated CRISPR repeats from the CRISPRCasdb database (version June 2019) to the six possible reading frames (translations with stop codons were discarded). Then, protein sequences with at least 2 matches from the 3 peptides of 1 strand, separated by 7–20 amino acids (equivalent to a distance 21–60 bp in DNA), were considered as potential spurious proteins (initial candidates). Both the gene name (locus_tag) and the genomic sequence with a minimal length of 500 kb (GenBank Accession Number) from these candidates were extracted, and *cas* genes were searched in a region of 10 kb both upstream and downstream. A total of 134 Position-Specific Scoring Matrices of Cas domains from the Conserved Domains Database (19) were used to search using RPS-BLAST from the BLAST 2.2.31+ package (20). The threshold to consider a Cas-positive protein was an E-value equal or lower than 1e-05, 25% identity and 70% domain coverage. A protein was considered as a potential spurious protein (putative false protein; PFP) when either a complete cluster of *cas* genes (all *cas* genes from one of the 33 subtypes classified in Makarova et al., 2020 (21)) or, at least, both *cas1* and *cas2* genes are found.

### Finding of spurious proteins searching for peptide repeats

To find spurious UniProtKB proteins originating from misannotated CRISPR sequences, we searched for proteins with perfect peptide repeats of length 7–20 amino acids, separated by spacers in the same length range. The hits were again evaluated by searching for *cas* genes around the corresponding candidate as described earlier.

### Cross-validation

Spurio was used to analyze all PFPs, using default parameters (22). This tool is based on a tblastn search and we considered it does not correctly predict the spurious protein when it does not find any hit in this initial similarity search, or the hit does not have a significant e-value, or the score of the final prediction does not exceed the default value.

## Results and discussion

### UniProtKB presents spurious proteins arisen from translated CRISPR sequences

**Figure 1.**
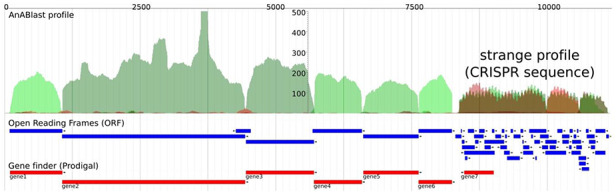
AnABlast profile of a genomic region that contains a CRISPR sequence (CP001172.2: 1 057 766–1 068 943). The profile shows accumulations of low-score sequence alignments between the genomic region and database protein sequences taken as protein-coding signals (called protomotifs). Green peaks correspond to accumulations in the forward strand, and red peaks correspond to accumulations in the reverse strand. Blue annotations represent ORFs, and red annotations are protein-coding genes predicted by a prokaryotic gene finder. Peaks above a height threshold, matching with predicted ORFs, stand for known protein-coding genes which are CRISPR-associated genes (*cas*) in this case (green peaks). But the strange profile at the end, which presents a series of both short peaks and ORFs in the two strands, including a putative gene in the reverse strand (gene7), is in fact a CRISPR sequence consisting of repeats (peaks) and spacers (valleys).

We previously developed a computational tool, AnABlast, for finding protein-coding sequences in whole genomes based on low-score alignments, called protomotifs, between the translation of a query genomic sequence and proteins from the UniProtKB database (23). The accumulation of protomotifs happens in regions that present protein-coding genes, but while analyzing a bacterial genome, we discovered a strange accumulation of protomotifs that turned out to be a CRISPR sequence (Figure 1).

**Figure 2.**
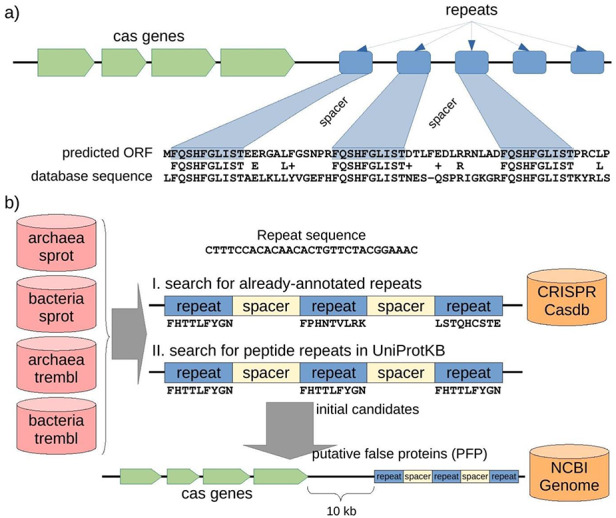
Structure of a CRISPR-Cas locus and its appearance when it is translated, and protocol to discover misannotated CRISPR sequences in protein databases. **(a)** A CRISPR-Cas locus includes a series of *cas* protein-coding genes followed by short nucleotide repeats surrounding heterogeneous sequences of a similar length called spacers. When a CRISPR sequence is erroneously translated, the corresponding amino acid sequence could present repeats separated by uniform spacers, and this protein would show similarity of 50% (centered in the repeat region) with other spurious proteins. **(b)** Searching for putative spurious sequences originating from translated CRISPR in four subsets of the UniProtKB protein database. The first approach (I) consisted in searching for translations of repeat sequences from the CRISPRCasdb database separated by putative spacers. The second approach (II) consisted in searching for amino acid repeats separated by putative spacers directly in the protein sequences. Finally, the initial candidates from the two approaches were mapped to their corresponding genomic sequences, and *cas* genes were searched within 10 kb around the candidate (see Methods for details). Proteins with both, repeats and *cas* genes nearby, are expected to be originating from the translation of spurious ORFs from CRISPR sequences, so called putative false proteins (PFP). Note that the first approach can take into account three different peptide sequences (drawn from the three possible reading frames of the nucleotide repeat sequence), while the second approach can only take into account one peptide sequence for all the possible repeats but can potentially discover new CRISPR repeats other than those already-annotated in the CRISPR database.

A CRISPR-Cas locus in prokaryotic genomes usually includes a cluster of *cas* genes followed by a number of sequence repeats of 20–60 bp in length with spacers of a similar length (Figure 2a). When several CRISPR sequences containing interspaced repeats are translated to amino acid sequences and stored in public databases, our gene finder will find protein alignments in which the sequence similarity is centered on the repeats.

To establish the extent of this contamination in current protein databases, we searched for translated CRISPR sequences in the UniProtKB database. To do this, we translated the repeats from a CRISPR database (CRISPRCasdb) and searched for clustered, regularly interspaced hits of these translations in archaeal and bacterial proteins (Figure 2b; see Methods for details). We found a large number of putative spurious proteins translated from CRISPR repeats (Table 1). This number was especially high in TrEMBL (the automatically annotated section of UniProtKB), though Swiss-Prot (the curated section of UniProtKB) also presented a few hits.

**Table 1.** Spurious proteins found in four subsets of UniProtKB when searching for already-annotated repeats. The table shows the number of sequences in each database section (sp = Swiss-Prot, tr = TrEMBL), the number of initial candidates, the average and standard deviation in the year of entry in UniProtKB database, the number of tested genomic sequences (after discarding genomic sequences lower than 500 kb), the number of candidates (and percent) with *cas* genes nearby, the number of candidates (and percent) with *cas* cluster nearby (PFP) and the number of tested candidates with Pfam domains (Cas(+), and PFP candidates in brackets).

	No. sequences	Initial candidates	Year (average)	Tested	Cas(+)	PFP	Pfam
**Archaea (sp)**	19 577	7	1998 ± 0	7	4 (57%)	4 (57%)	0 (0)
**Bacteria (sp)**	334 328	8	2006 ± 4	5	2 (40%)	2 (40%)	3 (0)
**Archaea (tr)**	3 950 817	348	2013 ± 4	295	204 (69%)	163 (55%)	4 (0)
**Bacteria (tr)**	129 646 170	6586	2015 ± 4	2417	1076 (45%)	901 (37%)	687 (3|0)
**Total**	133 950 892	6949	2015 ± 4	2724	1286 (47%)	1070 (39%)	694 (3|0)

**Figure 3.**
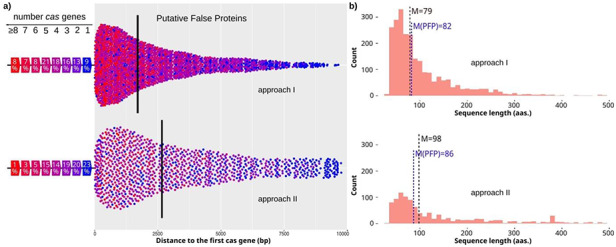
Characteristics of candidates with *cas* genes nearby. **(a)** Distances from candidates to the closest *cas* gene for approach I and II (top and bottom, respectively). The color of the dot shows the number of *cas* genes found close to the candidate. The percentage of candidates found close to a specific number of *cas* genes is shown on the left. Median value is shown by a vertical line. **(b)** Length distribution of candidates for approach I and II (top and bottom, respectively). The median value is highlighted for all candidates with one or more *cas* genes (M), and for the final PFPs (M(PFP) in blue color).

**Figure 4.**
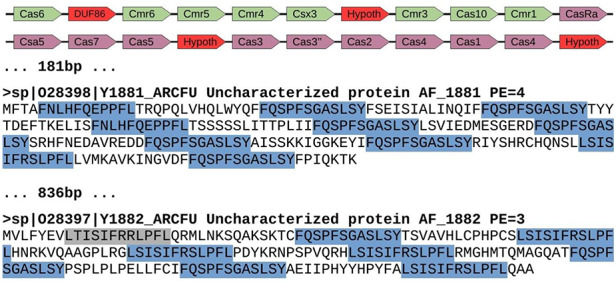
Genomic region of *Archaeoglobus fulgidus* with several spurious CRISPR sequences (AE000782.1: 1 671 367–1 694 202). This region contains a cluster of *cas* genes for CRISPR-Cas system class III-B (green color), followed by another cluster of genes for CRISPR-Cas system class I-A (purple color). They appear next to two spurious proteins with repeats arisen from three different reading frames. Red color shows uncharacterized or hypothetical proteins inside the *cas* clusters. Repeats are highlighted in blue color, with one of them in gray color because it represents a degenerated sequence. The distance between elements is shown in bp, and proteins from Swiss-Prot show the accession number, the identifier, the functional annotation (Uncharacterized protein), the gene name and the protein existence (PE), which represents the evidence that supports the existence of the protein (4 = protein predicted, and 3 = protein inferred from homology).

To assess these putative spurious proteins, we initially searched for independent *cas* genes, and later for complete *cas* clusters (or at least the pair cas1, cas2) in the surrounding genomic sequence (see Methods for details). Approximately 50% of the spurious proteins (1286 in total) has nearby one or more *cas* genes (1080 with complete *cas* clusters), and of those proteins, 78% were near 3 or more *cas* genes (Figure 3a). In addition, half of the spurious proteins have their *cas* genes within 2 kb. So, they constitute clear examples of misannotated proteins, which could be really non-coding CRISPR sequences (Figure 4; Suppl. Table S1). These proteins are primarily short peptides with a median length of 79 amino acids, which classify them as small ORFs with a length close to the ORFs found in randomized DNA (Figure 3b).

The other 50% of initial candidates that are not localized near any *cas* gene could constitute CRISPR sequences which have previously been called ‘split arrays’ because, despite not having *cas* genes nearby, they possess the same repeats as other CRISPR arrays (24). However, the candidates with greater certainty of being sequences originating from CRISPR sequences, which could be considered as spurious proteins, are those with a complete *cas* cluster, and hereinafter they will be referred as putative false proteins (PFPs) (Suppl. Table S2).

To determine whether the spurious sequences were part of particular protein families, we searched them for Pfam domains. We found that most sequences had no Pfam domains, and the ones that had them appeared almost exclusively in the non-*cas* candidates. Since Pfam domains usually constitute well-known protein families and 77% of UniProt protein sequences have a match to the Pfam database (18), candidates bearing these domains could be considered as true proteins not originating from CRISPR translations. However, three sequences with nearby *cas* genes, but not PFP, had also Pfam domains. Two of them have a DNA gyrase C-terminal domain (Pfam: PF03989) which is a short repeat appearing in tandem in gyrase proteins and is coincidentally similar to the CRISPR repeats of a strain of *Mycoplasma fermentans*. The other is a domain found at the N-terminus of many transposon-encoded proteins (Pfam: PF05598; DUF772).

It should be noticed that spurious proteins are coming not only from CRISPR sequences but also due to sequencing and assembly errors, or the translation of non-coding sequences. We wanted to compare our PFPs with those obtained by the tool Spurio, which is capable of discovering these types of sequences based on both nucleotide similarity and the occurrence of stop codons (22). Spurio found 789 out of the 1070 PFPs (Suppl. Table S2). This result suggests a cross-validation of both methods, but also shows that the current method can find new spurious sequences that could escape others. In this procedure, it is essential to have the most complete genomic sequence to allow the discovery of *cas* genes. For example, one of the clear spurious protein previously detected by Spurio is an uncharacterized protein from the bacterium *Acinetobacter bereziniae* (UniProt: N8YUQ2). It was also found by the current method (Suppl. Table S1), but it cannot be validated because its genomics sequence is only 864 bp long, which makes it impossible to find any *cas* gene.

### *Ab initio* search for proteins bearing spaced repeats barely discovers new spurious sequences

We have just found more than 1000 putative proteins with interspaced repeats which are identical to repeats in the CRISPRCasdb database and have *cas* genes nearby. But this approach would not find sequences originating from undiscovered CRISPR sequences which are not stored in CRISPRCasdb. To account for this, we searched for protein sequences bearing interspaced peptide repeats. Following this different approach, more than 50% of the initial candidates from the previous approach were found, and more than 467 000 new candidates were proposed (Table 2). Despite this large number of candidates, it should be noted that only proteins with repeats from one of the three possible peptides arisen from the original nucleotide repeat can be found using this protocol. If we had translated the three possible reading frames, we could have proteins with two different peptides separated by a spacer. Since we are not considering this kind of cases, the real number of candidates could be even greater.

**Table 2.** Spurious proteins found in four subsets of UniProtKB when searching for already-annotated repeats. The table shows the number of sequences in each database section (sp = Swiss-Prot, tr = TrEMBL), the number of initial candidates, the average and standard deviation in the year of entry in UniProtKB database, the number of tested genomic sequences (after discarding genomic sequences lower than 500 kb), the number of candidates (and percent) with *cas* genes nearby, the number of candidates (and percent) with *cas* cluster nearby (PFP) and the number of tested candidates with Pfam domains (Cas(+), and PFP candidates in brackets).

	No. sequences	Initial candidates	Previous	Tested	Cas(+)	PFP	New PFP	Pfam
**Archaea (sp)**	19 577	23	4(57%)	21	2(10%)	2(10%)	2	14(0|0)
**Bacteria (sp)**	334 328	506	6(75%)	381	0(0%)	0(0%)	0	346 (0|0)
**Archaea (tr)**	3 950 817	15 888	202(58%)	4026	170(4%)	110(3%)	15	2002(166|7)
**Bacteria (tr)**	129 646 170	450 640	4135(63%)	128 929	984(0.7%)	679(0.05%)	256	83 969(980|82)
**Total**	133 950 892	467 057	4347(55%)	133 357	1156(0.8%)	791(0.05%)	271	86 331(1146|89)

Although this approach found half of previous candidates and proposed many new ones, these new candidates rarely have *cas* genes nearby. In fact, only 271 new spurious sequences were validated by *cas* clusters nearby out of the 467 057 analyzed initial candidates (Suppl. Table S3). Therefore, most of these new candidates are expected to be real proteins. In fact, many of them show hits to domains from the Pfam database. These Pfam domains are mainly short amino acid repeats, showing a similar architecture to translated CRISPR sequences. One of these domains is the zinc-ribbon domain (Pfam: PF13240), which appears in archaeal RNA polymerase proteins (25) and presents a structure that is similar to a CRISPR sequence with two repeats (FCXXCG-15/17-FCXXCG). Interestingly, this domain appears in 67 candidates out of the 467 057 analyzed initial candidates, including one PFP originating from an uncharacterized protein of *Methanobrevibacter ruminantium* where the found repeat was GRGLFNKKT, while the Pfam domain is only found by chance (UniProt:D3E280_METRM).

In addition, candidates found by this approach have fewer *cas* genes close to them, and 43% of those show only 1–2 nearby *cas* genes (Figure 3a). Furthermore, their median length is again short (with a median of 98 residues), but they include a number of sequences with lengths around 400 amino acids, which again suggests that they are true proteins (Figure 3b). However, PFPs with complete *cas* clusters have a median length of 86, supporting their definition as spurious sequences.

Finally, the tool Spurio was used again with the PFPs, and now it only found 66 out of 271 spurious sequences unique to this approach II (Suppl. Table S2). Altogether, these results indicate that searching only by protein repeats could be a useful method to find CRISPR misannotated sequences in protein databases, though the large number of false positives found, together with the appearance of Pfam domains, suggest that it would be a non-specific method to discover spurious proteins. However, checking for the presence of proximal *cas* clusters seems to be an efficient way to discriminate for true CRISPR misannotated sequences. In summary, using this complementary approach, we have found 271 PFPs missed in the first screening and 205 of which were not detected by Spurio.

### The number of spurious proteins increases despite available computational tools for finding CRISPR sequences

**Figure 5.**
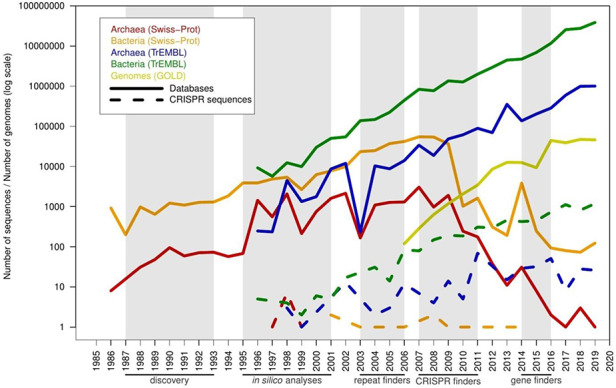
Timeline with the number of predicted spurious proteins by year of creation, compared with the pace of new proteins and genomes in the databases. The *x*-axis shows years from 1985 to 2019. A series of milestones are highlighted below the *x*-axis (and shaded in gray color): discovery of CRISPR sequences (discovery), initial computational analyses of these sequences (*in silico* analyses), prediction of CRISPR sequences using unspecific computational tools designed for searching tandem repeats (repeat finders), CRISPR-specific computational tools such as CRT and CRISPRFinder (CRISPR finders) and inclusion of CRISPR prediction in genomic gene finders such as Prokka, PGAP and RAST (gene finders). Solid lines represent database growth, and dashed lines represent misannotated sequences.

There are specific computational tools for finding CRISPR sequences since 2007. Therefore, spurious sequences in the protein database could be artifacts from the periods in which these tools were not available. To test this idea, the creation dates of the spurious candidates were compared with the database growth due to the increase in the rate of sequencing of genomes. The six PFP from the manually curated Swiss-Prot entered in this database just before the growth freeze of the database in 2010 (Figure 5). But the most important entry of spurious proteins precisely occurred starting from 2010, and it is parallel to the accelerated growth of TrEMBL and the sequencing of new archaeal and bacterial genomes. At the peak of entry of archaeal spurious sequences, many of the current CRISPR-specific finders had already been developed, such as CRISPRFinder/CRISPRCasFinder, PILER-CR and CRT (15, 26–28). And the peak of entry for bacteria occurred later, when gene finders had included CRISPR discovery in their algorithms, such as Prokka, RASTtk and PGAP (3, 29, 30), and higher accuracy would be expected in the annotation of CRISPR sequences. Currently, the entry of spurious proteins is not parallel to the database growth, probably because gene finders now correctly predict the majority of the CRISPR sequences.

UniProtKB is a database that feeds on different sources where the annotation tool used is also heterogeneous. Thus, we checked for the input source of these spurious proteins and found that all the nucleotide sequence databases provide this type of sequences: GenBank (778), EMBL (432) and DDBJ (126). The gene prediction method was also different, and 174 spurious sequences were annotated by Prodigal, 136 by AMIGene and 22 by GeneMarkS+ (Suppl. Table S2).

## Conclusions

Translated CRISPR sequences are contaminating protein databases despite current gene finders that specifically predict this kind of elements in bacterial and archaeal genomes. These spurious proteins are coming from heterogeneous annotation sources, so all gene finders should be reviewed to avoid this problem. Remarkably, these spurious proteins could affect later secondary analyses, such as the prediction or annotation of new sequences originating from complete genomes, and we suggest the database to remove them to avoid potential harmful consequences.

We propose a new protocol to uncover these spurious sequences. It involves the comparison of protein sequences against a database of CRISPR repeats, and subsequent *cas* genes finding, since the direct search for amino acid repeats in the protein sequences can find short domains that, while having a similar structure to spuriously translated CRISPR repeats, can happen in *bona fide* proteins.

In conclusion, we analyzed more than 460 000 candidate spurious proteins and propose removing 1341 of them from the database (Suppl. Table S2). We also suggest that new prokaryotic protein sequences should be tested according to our protocol before their entry in public protein databases.

### Highlights

- Translated CRISPR sequences are contaminating protein databases.
- Translated CRISPR sequences usually form short proteins.
- New annotated protein sequences should be checked when presenting interspaced repeats and *cas* clusters nearby.

## Supplementary Material

baaa088_Supp

## Data Availability

Scripts are available in the GitHub repository together with links to tables with the raw results: https://github.com/UPOBioinfo/crispr_spurious/
